# Vaccines against Tuberculosis: Where Are We and Where Do We Need to Go?

**DOI:** 10.1371/journal.ppat.1002607

**Published:** 2012-05-10

**Authors:** Tom H. M. Ottenhoff, Stefan H. E. Kaufmann

**Affiliations:** 1 Department of Infectious Diseases, Leiden University Medical Center, Leiden, The Netherlands; 2 Deptartment of Immunology, Max Planck Institute for Infection Biology, Berlin, Germany; International Centre for Genetic Engineering and Biotechnology, India

## Abstract

In this review we discuss recent progress in the development, testing, and clinical evaluation of new vaccines against tuberculosis (TB). Over the last 20 years, tremendous progress has been made in TB vaccine research and development: from a pipeline virtually empty of new TB candidate vaccines in the early 1990s, to an era in which a dozen novel TB vaccine candidates have been and are being evaluated in human clinical trials. In addition, innovative approaches are being pursued to further improve existing vaccines, as well as discover new ones. Thus, there is good reason for optimism in the field of TB vaccines that it will be possible to develop better vaccines than BCG, which is still the only vaccine available against TB.

## Introduction

It has proven challenging to develop vaccines against pathogens whose control depends on the cellular immune response. Tuberculosis (TB) is no exception. Most successful vaccines today target pathogens against which humoral immunity suffices to achieve protection and often sterile eradication. But TB vaccines need to drive primarily the cellular arm of the immune system. *Mycobacterium tuberculosis (Mtb*), the intracellular pathogen that causes TB, was discovered in 1882 by Robert Koch (Nobel Laureate in 1905) and is responsible for more human deaths than any other single pathogen today [1]–[3]. The combination with HIV co-infection, which dramatically compromises host resistance to TB, leads to high disease prevalence in affected endemic populations. Currently, each year more than 1.5 million people die of TB, and more than 9 million newly develop TB [4]. This dire situation is further compromised by the increasing prevalence of multidrug-resistant (MDR) and extensively drug-resistant (XDR) *Mtb* strains [5], and the more recent occurrence of TDR (totally drug-resistant) *Mtb* strains, which are virtually untreatable [6].

TB, known also as the white plague, has been around for millennia. Early last century, hopes were that TB could be conquered by vaccination with the newly developed *M. bovis* BCG vaccine, isolated by and named after Calmette and Guérin in Lille, France [7]. These hopes were further boosted by the development of the first anti-tuberculous drugs during WWII by Selman Waksman (Nobel Laureate in 1952), who discovered that streptomycin was bacteriostatic for *Mtb* [8]. Initially, treatment with streptomycin appeared highly efficacious, but the tide turned when drug resistance rapidly developed, an early testimony of *Mtb*'s ability to acquire drug resistance when treated by single antibiotics. Despite this early writing on the wall, the misconception that TB could be conquered by antibiotics and BCG vaccination led to complacency for several decades. This situation dramatically changed only in the early 1990s, when the World Health Organization (WHO) declared TB a global emergency. From that time onwards TB scientists, who had been focusing much of their efforts on other areas of research and development due to a lack of interest in and funding for TB, were able to reorient efforts and initiate significant activities in the study of TB [9]. Soon, researchers determined *Mtb*'s genome sequence [10], and began to dissect TB's immunology and cell biology.

Although in most cases, TB can be treated successfully with multidrug combinations of antibiotics (except for MDR-, XDR-, and TDR-TB), treating TB cases is clearly insufficient to interrupt disease transmission in highly endemic populations, because an active TB case will typically infect some 10 to 15 contacts [4] per year. Better preventive measures that block *Mtb* transmission need to be developed, including vaccines that prevent establishment of *Mtb* infection in the susceptible human host (infection-blocking vaccines), or alternatively, vaccines that prevent progression of established infection towards active TB disease (disease-progression-blocking vaccines). The only available vaccine against TB today remains the almost one-century-old BCG. It is routinely administered to infants in many countries worldwide and provides significant protection against severe forms of TB, mostly disseminating and meningeal forms. However, the protective efficacy of BCG against pulmonary TB in adults is inconsistent and incomplete, and BCG vaccination campaigns have had little impact on the occurrence of pulmonary TB, which represents the transmissible form of this disease. It is still unclear why the protective effect of neonatal or early-age BCG vaccination often begins to wane in early adolescence, at least in TB-endemic areas, as will be discussed further below. Nevertheless, in a North American study, BCG's protective efficacy was found to last for over 50 years [11], thus pointing to the importance of possible environmental modulators (co-infections, co-morbidity, nutrition, genetics, TB exposure intensity, etc.; see below). Another issue of concern that compromises BCG's utility is that infants with HIV have an increased risk of developing disseminated BCG-osis [12], bearing similarities to individuals with genetic deficiencies in the interleukin (IL)-12/IL-23/interferon-gamma (IFNγ)/signal transducer and activator of transcription (STAT)1 pathway [13], [14]. This implies that TB vaccines need to be developed that not only have a superior ability to induce protective immunity against TB, but also have a better safety profile compared to BCG [12], [15].

There has been significant progress in TB research over the last 20 years. From a pipeline empty of new TB vaccine candidates in the early 1990s, more than 12 novel TB vaccine candidates now have been or are being evaluated in clinical trials in humans. Moreover, many new approaches and strategies are under evaluation for further improvement of these and other vaccines. This raises hope that vaccines can be developed which are better than BCG. It will be critical to mobilize the needed funding, political, and supranational support, and increase public awareness of the pressing needs in TB in order to step up efforts towards controlling this resilient disease. The recent declaration by the European Parliament to support TB vaccine research and development (http://www.tbvi.eu/news-agenda/news/news-message/eu-parlement-discusses-the-importance-of-tb-vaccines.html) is a laudable step in this direction.

## TB Infection Control

Infection with *Mtb* does not necessarily lead to TB disease. Only 3%–10% of immunocompetent individuals that are infected will progress towards active disease during their life-time [16]. It is unknown if—and what proportion of—infected individuals are able to possibly achieve complete or “sterile" eradication of bacteria following primary pulmonary infection. Existing yet indirect evidence suggests that this may be the case and could be dependent on innate rather than adaptive immune defense mechanisms, as persons have been described who despite high intensity exposure—and thus likely infection—remain tuberculin skin test (TST)-negative and do not develop disease [17], [18]. The TST is a classical indicator of delayed type hypersensitivity-dependent T-cell activity towards *Mtb* antigens in the case of previous contact. TST surveys suggest that around one-third of the world's population, amounting for over 2.2 billion people, is infected with *Mtb* [4]. Although >90% are able to contain infection in a latent or subclinical stage, this huge reservoir of latently infected individuals fuels the high numbers of new active TB cases [19]. The development of active TB is dramatically accelerated by co-infection with HIV, which increases *Mtb* reactivation rates from 3%–10% per life-*time* to 5%–10% per life-*year* [20]. Latent *Mtb* infection also poses iatrogenic clinical challenges, due to increases in TB reactivation following administration of biologicals, such as tumor necrosis factor-alpha (TNFα)/IL-12/IL-23 blockers in treating inflammatory diseases like rheumatoid arthritis, Crohn's disease, and psoriasis [21]. New diagnostic tests that distinguish more specifically between *Mtb* infection and BCG vaccination or infection with non-tuberculous mycobacteria (NTM) than the classical TST have been developed recently, based on *Mtb* antigen-specific release of IFNγ in whole blood or isolated leucocytes. These assays have been coined interferon gamma release assays (IGRAs) and have utility in clinical management, although sensitivity issues in borderline IGRA-positive and in remotely infected persons, which may result in false-negative test results, still need to be addressed [22].

A precise understanding of protective immunity and protective host defense is still needed. The study of individuals who are able to control *Mtb* infection in the long term may be particularly informative in this respect. Despite two decades of intensified research, we have to acknowledge that we still do not fully understand what exactly constitutes protective immunity and host defense against TB (see below section on TB immunology). If we could decipher essential mechanisms, pathways, and biomarkers of protection, then pathways of host defense could be targeted by rationally designed vaccines, and protection measured by accurate biomarkers [23], [24]. Obviously, TB is much more complex than, for example, many viral infections, in which highly pathogen-specific mechanisms and correlates of protection exist in the form of neutralizing antibodies. An additional challenge in TB is the need to do better than achieve natural immune control of long-term infection: notably, by inducing immune responses that not only mediate life-long control of *Mtb* infection (which poses the inherent risk of endogenous reactivation), but also achieve sterile eradication of bacteria from the infected host. First-generation new TB vaccines in clinical trials are designed to achieve stronger immunity but may not necessarily succeed at sterile eradication (discussed below) [25], [26].

Besides mediating protection on its own, the host immune response is also known to synergize with chemotherapy through incompletely defined mechanisms [27]. Thus not only preventive, but also therapeutic, TB vaccines that improve the host response to chemotherapy will be of significance; the latter may help shortening treatment, as well as reducing and perhaps treating drug resistance.

## *M. bovis* BCG: The Current TB Vaccine

BCG is the one of the most widely administered vaccines worldwide, having been given over 4 billion times. BCG has been part of the expanded program on immunization (EPI) since the early 1970s and features relatively few serious adverse events. Only fairly recently has it become apparent that individuals with genetic defects in key immune genes or infants with clinically active HIV infection are highly susceptible to developing disseminating BCG disease, posing a significant risk in HIV-burdened populations where TB is often highly endemic [12]. This has led to changes in policy from the WHO Global Advisory Committee on Vaccine Safety, recommending that BCG should not be used in HIV-positive children.

Despite the relative efficacy of BCG in infants, the major unanswered question is why BCG fails to prevent pulmonary TB in adolescents. It has been proposed that immune memory wanes in adolescence, which is the most critical period for TB infection and/or its progression to active disease. The fact that immunological memory is induced by BCG at an early age (neonates or infants) at which the immune system is not yet fully mature may provide one explanation. However, other factors may also contribute to decreasing BCG efficacy and/or enhanced susceptibility of young adults to TB. These factors include co-infections with helminths; viruses that compromise host immunity (an obvious, extreme example is HIV); and NTM that can induce immune regulation [28]. Helminth infection has been associated with increased TB incidence and reduced BCG vaccine efficacy in affected populations [29]. Such modulatory effects may be mediated by immune deviation, e.g., the induction of T helper 2 (Th2) or regulatory T-cell (Treg) responses, which dampen the protective efficacy of Th1-dependent protection. A recent study in mice documented that helminth infection can impair innate pulmonary host defense against *Mtb* through the IL-4 receptor pathway [30].

Whatever the explanation for its inadequacy, BCG is clearly insufficient for worldwide TB control. Thus, there is a strong need to develop vaccines that can either boost BCG's initial priming and protective effects, or replace BCG by superior vaccines. Both strategies and approaches will be discussed below, after a short introduction to the key immunological features of TB.

## The Immunology of TB

**Figure 1:**
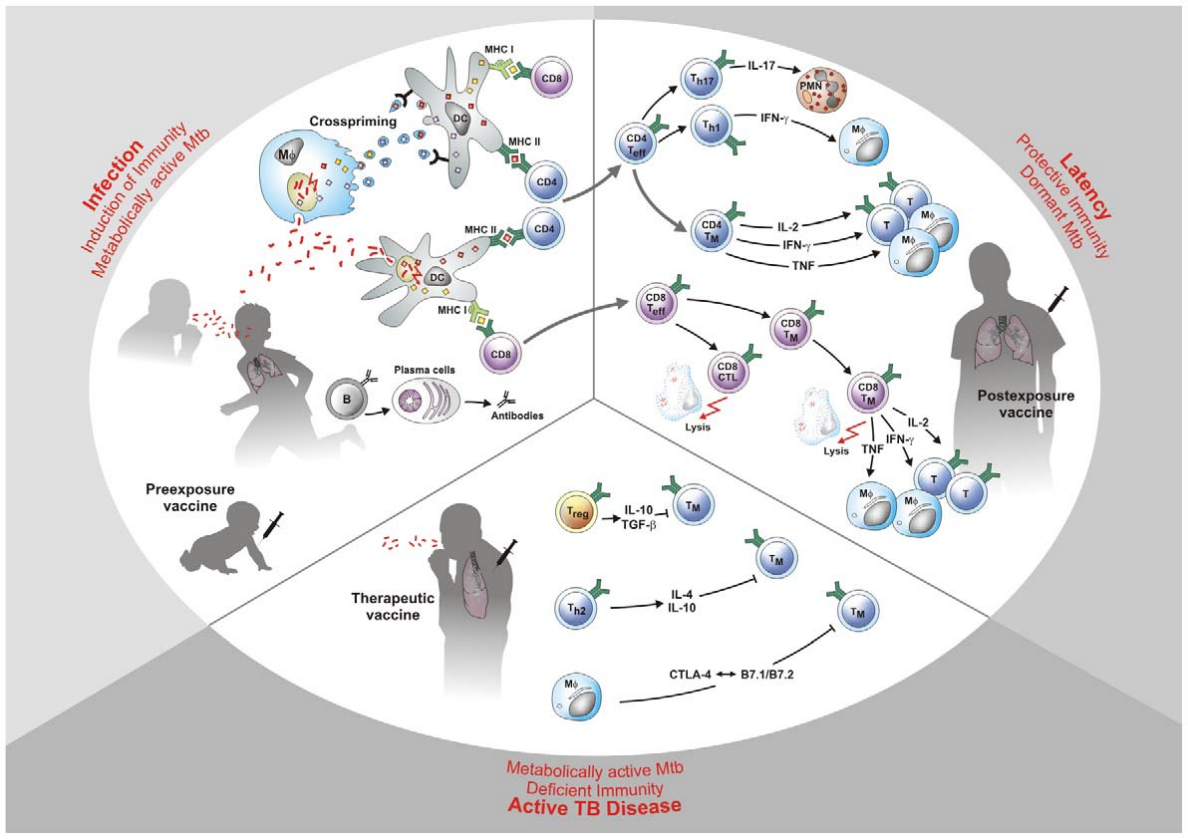
The three stages of tuberculosis. **Stage 1**: Infection of *Mycobacterium tuberculosis* (*Mtb*) frequently occurs at a young age. Metabolically active *Mtb* are inhaled and subsequently T-cells are stimulated which carry the major burden of acquired immunity. These include major histocompatability complex class II (MHC II)-restricted CD4 T-cells and MHC I-restricted CD8 T-cells. B cells are also activated but their protective role in TB remains elusive. Pre-exposure vaccines are given at this early stage. Novel pre-exposure vaccine candidates are given very soon after birth and thus generally before infection with *Mtb*. They either substitute for Bacille Calmette Guérin (BCG) or boost immunity induced by BCG. **Stage 2**: Acquired immunity comprising CD4 and CD8 T-cells contains *Mtb* in a dormant stage within solid granulomas. T-cells produce type I cytokines and cytolytic effector molecules. They become memory T-cells which concomitantly produce multiple cytokines. Individuals remain latently infected without clinical signs of active tuberculosis (TB). Post-exposure vaccines are given to adolescents or adults who are latently infected but healthy. **Stage 3**: Mechanisms leading to deficient immunity and disease reactivation are numerous and include production of suppressive cytokines such as interleukin (IL)-10 and transforming growth factor-beta (TGFβ) by T helper 2 (Th2) cells and regulatory T(reg) cells as well as T-cell exhaustion mediated by inhibitory receptor-coreceptor interactions on antigen presenting cells (APCs) and T-cells. *Mtb* becomes metabolically active and granulomas become caseous. *Mtb* can be spread to other organs and to other individuals. Therapeutic vaccines are given to TB patients in adjunct to chemotherapy.

*Mtb* organisms are transmitted by aerosols originating from the lungs of persons with active TB. *Mtb*-loaded aerosols are inhaled by TB contacts and reach the alveoli of their lungs, where they are taken up by various cell types, including alveolar macrophages, interstitial macrophages, local dendritic cells (DCs), and likely also epithelial cells (Figure 1: Infection). In humans and mice, *Mtb* infection is followed by a delayed onset of adaptive immune responses when compared to other infections [31]. Recent studies in mice have shown that *Mtb* possesses the unique ability to establish infection while delaying the onset of an adaptive immune response by 2–3 weeks. Using animals carrying genetically engineered T-cell receptors specific for *Mtb* antigens, it has been shown that priming of specific T-cell responses against *Mtb* takes place only in the local draining lymph nodes of the lung, and that transport of *Mtb*-infected cells from the lung parenchyma to the lymph nodes is significantly delayed for a period of at least 1–2 weeks [32]–[36]. It remains unknown which cell types are involved in *Mtb* antigen presentation to naïve T-cells, either DCs that cross-present antigens from infected cells, or perhaps also DCs migrating from the lung [37]. It is thought that *Mtb* can actively inhibit the migration of infected cells from the lung to the draining lymph nodes, or that *Mtb* can infect cells with only a poor migratory potential like epithelial cells. In both cases, however, initiation of *Mtb*-specific T-cell immunity is delayed. This “procrastination" strategy likely allows *Mtb* to establish infection and build up a critical mass of infecting organisms, from which two types of populations can be derived: (i) “dormant", “nonreplicating", or persisting organisms that remain in a metabolic low activity state in infected cells, and present a potential reservoir of bacteria that can resuscitate later on in life (Figure 1: Latency); and (ii) actively replicating metabolically active bacteria that stimulate the host immune response, which confers protection but can also precipitate TB pathology. The latter is hallmarked by the formation of central caseous necrotic lesions, filled with extracellular infectious *Mtb* and cell debris [38].

*Mtb* is able to persist inside otherwise hostile host cells, notably professional phagocytes, by employing different immune evasion strategies. These will not be discussed here in detail, but include the inhibition of phagosome maturation, the inhibition of autophagy, the inhibition of apoptosis, egression into the cytosol, blocking of MHC antigen processing and presentation, and the inhibition of IFNγ-receptor signaling, all of which help *Mtb* to evade host defence [39]–[43].

Following initial deposition of *Mtb* in the lung, macrophages and other immune cells are recruited during the early innate response to infection. The resulting cellular infiltrates become organized as primary granulomas. These are highly dynamic structures from which cells can rapidly in- and efflux. At a later stage, when adaptive immunity has been initiated, specific T-lymphocytes start infiltrating the granuloma; this leads to the formation of larger, well-organized, solid granulomas in which *Mtb* organisms typically are located mostly centrally. If control of infection and inflammation is not balanced optimally, the central regions of the granulomas will become necrotic and later caseous as just discussed [44], allowing outgrowth of high numbers of *Mtb* organisms. When these liquefying granulomas also damage airway linings, infected material is discharged into the airways. At this stage, TB infection has become contagious (Figure 1: Active TB disease).

Thus immunity to *Mtb* is a two-edged sword: it protects the human host against disseminating infection, but also facilitates transmission of TB to contacts. Therefore, TB vaccines not only need to induce optimal immunity to *Mtb*, but also a balanced response that favors protective and avoids pathogenic mechanisms.

The *Mtb*-specific immune response comprises a multitude of different cell types. This ranges from classical and non-classical T-cells to neutrophils, B-cells, and natural killer (NK) cells. The role of CD4^+^ Th1-cells in TB is best understood. Th1-cells, which secrete IFNγ and TNF as signature cytokines, are crucial for protective immunity against mycobacterial infections. Their induction is strictly dependent on the secretion of phagocyte-produced IL-12 [13]. Deleterious mutations in genes encoding proteins essential to the induction (IL-12p40 subunit, IL-12Rβ1) or function (IFNγR1, IFNγR2, Stat1) of Th1-cells, or their depletion due to HIV infection, greatly enhance susceptibility to severe infections due to otherwise poorly pathogenic mycobacteria or *Mtb* [13], [45]. This is also highlighted by the enhanced incidence of TB reactivation during treatment with biologicals targeting TNF, which are used in treating inflammatory disorders such as rheumatoid arthritis, Crohn's disease, and psoriasis [21].

CD4^+^ Th1-cells recognize *Mtb* antigens presented by MHC-class II molecules on DCs and macrophages [46]–[48], the dominant habitats of *Mtb*. As mentioned above, CD4^+^ T-cells are initially primed in the draining lymph nodes of the infected lung. Endosomal/phagosomal antigens like *Mtb* naturally access the MHC-class II processing route, which leads to the presentation of *Mtb* peptide-loaded MHC-class II complexes, providing “signal 1" at the DC surface. Only in the presence of a second signal, which is provided by co-stimulatory molecules, such as CD80, CD86, CD40, CD27, and 4-1BB/CD137, and a third signal, provided by essential cytokines like IL-12, however, full activation and differentiation of Th1-cells takes place. In the absence of any of these three essential components, T-cell activation may either not be initiated, or be preliminarily aborted; e.g., in the absence of signal 2, T-cell tolerance/anergy can result. Recent studies suggest that T-cell activation in TB granulomas is far from optimal but the limiting factors, which may be antigen/MHC-class I/II availability, lack of co-stimulation, or lack of supportive cytokines, still need to be elucidated [49].

Next to CD4^+^ Th1-cells, more recent evidence supports the prominent activation of Th17-cells in TB. This is a recently discovered T-cell subset that produces IL-17A, IL-17F, in many cases IL-22 and TNF, and in some cases also IFNγ. Th17-cells are pro-inflammatory and mediate anti-microbial immunity against extracellular bacteria and fungi, particularly at mucosal surfaces. The exact mechanisms leading to Th17 activation and differentiation are less clear than for Th1-cells but are believed to involve phagocyte-dependent production of IL-1β or IL-6, and transforming growth factor-beta (TGFβ). Other studies have pointed towards the essential role of IL-23, a closely related family member of IL-12, in the induction and expansion of Th17-cells or Th17 memory cells [50]. Current evidence suggests that Th17-cells play a significant role in the early phase of protection against high-dose aerosol *Mtb* infection in mice [51], [52]. Th17-cells emerged in the early phase of adaptive immunity and contributed to host defense against intracellular pathogens, including *Mtb* [53]: IL17A has been found to be essential in forming mature granulomas in the lung following BCG or virulent *Mtb* infection [54]. Their role during chronic and latent infection is less clear [55]: hyperactivity of Th17-cells in previously *Mtb*-infected animals induced by multiple BCG revaccinations was detrimental and led to increased immunopathology with IL17-MIP2-dependent influx of neutrophils and tissue destruction rather than containment of infection in the lung [55], [56]. The major source of early IL-17 during infection, however, may not be from CD4^+^ Th17-cells but rather from T-cell receptor gamma delta (γδ) cells [54], [56].

In addition to CD4^+^ T-cells, CD8^+^ T-cells also contribute to optimal immunity and protection against TB [48], [57]. The mechanisms underlying CD8^+^ T-cell activation in TB are incompletely defined. It is clear that DCs possess multiple pathways to load MHC-class I molecules, either by classical cytosolic processing, or by alternative processing of phagosome-located pathogens and endosome-located antigens. Active transmembrane transport, or leakage through micro-damaged membranes of *Mtb* phagosomal antigens to the classical cytosolic proteasome/MHC-class I presentation pathway, may also lead to MHC-class I loading with *Mtb* peptides. The recent suggestion that virulent mycobacteria can escape from the phagosome into the cytoplasm and thereby directly access MHC-class I processing/presentation may provide a new mechanism [43]. Thirdly, *Mtb*-infected cells can undergo apoptosis, leading to the formation of apoptotic vesicles that are taken up by DCs, after which the antigenic cargo is cross-presented through MHC-class I and class II molecules [58], [59]. Fourthly, autophagy, which plays a prominent role in cellular homeostasis and in bacterial sequestration in vacuolar organelles, is involved in antigen presentation to, and cross-priming of, T-cells in the response to intracellular pathogens, including *Mtb* [60], [61]. Thus, there are multiple pathways for activation of CD8+T-cells by phagosomal antigens.

Besides classical MHC-class Ia-restricted CD8^+^ T-cells, also MHC-class Ib-restricted CD8^+^ T-cells have received attention recently [62], [63] . These include HLA-E-restricted cells [64], [65]; lung mucosal-associated invariant T-cells (MAIT) that recognize *Mtb* antigens in the context of the non-classical molecule MR1 [66]; and CD1-restricted T-cells that mostly recognize lipid antigens derived from *Mtb*. CD1b molecules follow the MHC-class II presentation route, but relatively little is known for MR1 and HLA-E. HLA-E molecules are enriched in the *Mtb* phagosome, thus providing a plausible mechanism for *Mtb* peptide loading [66].

CD8^+^ T-cells are endowed with multiple mechanisms to attack *Mtb*-infected cells. Importantly—and in contrast to CD4^+^ Th-cells—CD8^+^ T-cells are able to recognize non-phagocytic cells such as epithelial cells, which can also be infected by *Mtb* [67]. All nucleated cells express MHC-class I molecules while only professional phagocytes—or IFNγ-activated cells—express MHC-class II molecules and can be recognized by CD4^+^ T-cells. Thus CD8^+^ T-cells are able to survey larger numbers of cells and a broader range of cell types compared to CD4^+^ T-cells. In addition, as already mentioned above, CD8^+^ T-cells are able to survey the cytoplasmic compartment of infected cells for pathogen-derived peptides through various antigen processing/presentation routes. Thus CD8^+^ T-cell immunity offers clear advantages and complementarity to CD4^+^ T-cell immunity.

CD8^+^ T-cells are able to secrete granules that contain cytotoxic molecules such as perforin, granzymes, and granulysin. These molecules can lyse host cells, and in the case of granulysin, also directly kill *Mtb* and other bacteria. In addition, CD8^+^ T-cells can induce apoptosis of infected target cells through ligating Fas or other TNF-R family-related cell-death receptors. Apoptosis may be activated to control *Mtb* infection (and is also counteracted by virulent *Mtb* [68]. Thirdly, CD8^+^ T-cells are also able to release Th1 cytokines such as IFNγ, TNF, and in many cases also IL-2. These different effector pathways have been well documented in humans [69]–[75]. These functions are also probably shared by MHC-class Ib-restricted CD8^+^ T-cells, suggesting a role in protection for classical as well as non-classical CD8^+^ T-cells in TB.

Besides T-cells, new evidence also suggests accessory roles for NK-cells and B-cells in optimal immunity against TB [76]. Emerging evidence strongly suggests that the activation and infiltration of granulocytes is associated with TB disease activity and may play an important role in TB pathogenesis [77], [78]. Recent genome-wide gene expression studies in TB patients revealed a striking neutrophil-associated expression pattern that was dominated by a type-1 interferon signaling signature. This profile normalized after curative treatment of infection, and had a core *Mtb*-specific component. Thus, other cells certainly contribute to host defense in TB, and may provide new biomarker signatures of pathogenesis, disease activity, and protective immunity.

Despite the more limited knowledge on the role of CD8^+^ T-cells in TB compared to CD4^+^ T-cells, paradoxically there seems to be greater consensus on “multifunctionality" as a marker of protection in TB for CD8^+^ T-cells than for CD4^+^ T-cells. Multi- or poly-functionality is a term used to describe the simultaneous production of multiple cytokines (mostly IFNγ, IL-2, TNF) or the expression of multiple effector functions (perforin, granulysin, cytolysis, etc.) by one single T-cell clone. For example, the co-expression of IFNγ and IL-2 by *Mtb*-specific CD8^+^ T-cells was associated with protective host defense following curative TB treatment [70], [79]. Conversely, there is some controversy whether poly-functional CD4^+^ T-cells are associated with protection, or even with disease. Some reports, mostly derived from vaccination studies in animals, have suggested that poly-functional (IFNγ^+^IL-2^+^TNF^+^) CD4^+^ T-cells are associated with protective immunity [80], [81], but more recent studies have suggested that these cells may not indicate protection, and even mark TB disease activity [82]–[84]. This issue clearly needs further investigation. In this context, it needs to be realized that even during active TB disease not all lesions in the human lung are reactivating. At least some sites of infection are probably still controlled by the host immune response. Thus, much akin to recent new models that describe latent TB as a graded continuum of infection rather than as a single static entity [85], it is likely that TB disease follows a similar spectrum, ranging from the reactivation of only few lesions up to full-blown activation of many lesions such as seen in rapidly disseminating disease. It follows that in mild and less severe forms of TB, in which only part of lesions is reactive, others may be adequately controlled by the host response, which may involve multi-functional CD4^+^ T-cells. In any case, further studies to resolve these controversies are eagerly awaited.

Besides T-cells endowed with protective effector functions, *Mtb * also induces activation and expansion of regulatory T-cell (Treg) populations. These range from naturally occurring, broadly reactive CD4^+^CD25^+^FoxP3^+^ Tregs, to antigen-specific-induced CD4^+^ Tregs [86], [87] and newly identified CD8^+^ Tregs, which have been mostly identified in the human response to mycobacteria [64], [88], [89]. These Treg have multiple inhibitory effects: they can directly suppress CD4^+^ Th-cell activity through secretion of IL-10, CCL4, or the expression of membrane-bound TGFβ; they can deactivate APCs and thus inhibit optimal priming of CD4^+^ responses; and they can inhibit the influx of CD4^+^ T-cells into draining lymph nodes, and/or inhibit their proliferation and expansion [86], thus delaying and inhibiting onset of adaptive immunity to *Mtb*.

Thus a multitude of interlinking cells and mechanisms are involved in the immune response to *Mtb* infection, including activating/effector, and inhibitory/regulatory functions. The balance of the response and the ability to deliver effective hits to *Mtb*-infected target cells dictates whether the host response will lead to control of infection and possibly bacterial eradication, or whether infection will progress with increasing numbers of bacteria, lung tissue damage, and transmission of *Mtb* organisms to new susceptible hosts [44].

## Designing Better TB Vaccines: Two Basic and Complementary Approaches

In the absence of reliable TB biomarkers of protection and a human challenge model, TB vaccine design by necessity will have to rely on empirical testing of candidate vaccines in long and costly efficacy trials. The identification of protective biomarker signatures would greatly facilitate vaccine discovery and testing [23], [24]. If it were possible to identify a correlate with the same potency as neutralizing antibodies against viruses, discovery of high profile TB vaccine candidates and clinical testing could be markedly accelerated. As of 2009, more than a dozen TB vaccine candidates entered clinical trials, and many more are in the pre-clinical pipeline to be considered for testing in phase I clinical trials [3]. Five vaccines are currently in phase I/IIa (safety/immunogenicity) clinical trials and two of these are in larger phase II clinical trials. At least 16 vaccine candidates are in advanced pre-clinical development and well over 20 next-generation candidates are in the discovery pipeline.

The development of new TB vaccines follows two basic avenues [1], [25], [90]. The first aims at replacing BCG by either improved recombinant (r)BCG or by genetically attenuated *Mtb*. The profile of genetically improved rBCG should be: a) safer; b) more immunogenic; c) inducing longer lasting protection; and d) inducing protection against highly virulent clinical isolates such as *Mtb* Beijing strains, as well as MDR, XDR, and TDR *Mtb* strains. One way to improve BCG is either by introducing immunodominant *Mtb*-specific antigens that are absent from BCG, such as RD1 locus-encoded antigens (ESAT6, CFP10); or by over-expressing antigens that BCG already expresses by itself (cognates of Ag85 complex), but probably not sufficiently high throughout all phases of infection. Another way to improve BCG is by introducing genetic modifications for superior targeting of essential immune pathways, for example, by enhancing or facilitating cross-priming; and to inhibit its ability to neutralize phagosomal maturation. Obviously, both strategies can be combined [38]. Besides these two rBCG vaccine approaches, a second major strategy to develop better live vaccines against TB has been to attenuate *Mtb*. These strategies include the deletion of essential metabolic genes to create auxotrophic mutants; or the deletion of major virulence genes and their regulators (see below). In either case, at least two independent genetic loci need to be deleted to avoid reversion to virulence. One study engineered *M. microti* to express RD1 *Mtb* antigens, which indeed resulted in better experimental protection [91]. A recently published third type of live mycobacterial vaccine against TB is recombinant *M. smegmatis*. When a locus called *esx-3* was removed from the *M. smegmatis* genome, a strongly enhanced innate immune response was seen. Unexpectedly, when this attenuated strain was complemented by the transfer of the *Mtb esx-3* locus, strong bactericidal and protective activity against *Mtb* was observed in the mouse model, and in some cases even leading to sterile eradication [92].

The second major avenue to develop better TB vaccines relies on the development of subunit vaccines. These are non-live, or in the case of viral vectors, non-replicating vaccines, which can be delivered safely into the human host regardless of immunocompetence. Subunit vaccines in TB are mostly based on recombinant proteins admixed with proper adjuvants, or the use of attenuated viral vectors. Although subunit vaccines theoretically could be used as priming vaccines, current views are that they may be mostly used as booster vaccines on top of BCG-, recombinant BCG-, or attenuated *Mtb*-priming vaccines. The subunit vaccines are then anticipated to boost strong, long-lived immune responses in already primed individuals, such that these will persist to levels high enough to protect the 3%–10% vulnerable individuals against TB disease.

The various types of vaccines and their intended use are summarized in Table 1.

**Table 1:** Most advanced TB vaccine candidates in clinical trials.

Type	Candidate	Description	Clinical Trial Status
Recombinant BCG for pre-exposure prime vaccination	VPM 1002	rBCG-expressing listeriolysin and urease deletion	Phase IIa ongoing
	rBCG30	rBCG-expressing Ag85B	Phase I completed/on hold
	Aeras-422	rBCG-expressing perfringolysin and Ag85A,85B, Rv3407	Phase I terminated due to side effects
Viral-vector for pre-exposure booster vaccination	Oxford MVA85A/Aeras-485	Modified vaccinia Ankara-expressing Ag85A	Phase IIb ongoing
	Crucell Ad35/Aeras-402	Replication-deficient adenovirus 35-expressing Ag85A, Ag85B, TB10.4	Phase IIb ongoing
	AdAg85A	Replication-deficient adenovirus 5-expressing Ag85A	Phase I
Fusion protein in adjuvant for pre-exposure booster vaccination	Hybrid 1+IC31	Fusion of Ag85B and ESAT-6 in adjuvant IC31	Phase I, soon entering IIa
	Hybrid 56+IC31	Fusion of Ag85B, ESAT-6 and Rv2660c in adjuvant IC31	Phase I ongoing
	Hybrid 1+CAF01	Fusion of Ag85B and ESAT-6 in adjuvant CAF01	Phase I ongoing
	M72+AS01 or AS02	Fusion of Rv1196 and Rv0125 in adjuvant AS01 or ASO2	Phase IIa ongoing
	Aeras-404: HyVac4+IC31	Fusion of Ag85B and TB10.4 in adjuvant IC31	Phase I
Whole bacterial vaccine for therapeutic vaccination	RUTI	Detoxified *M. tuberculosis* in liposomes	Phase IIa ongoing
	*M. vaccae*	Inactivated *M. vaccae*	Phase III completed

### Live Vaccines, Aiming to Replace BCG as Priming Vaccines

The first attempts to create improved BCG vaccines were made by Horwitz et al., who over-expressed antigen 85B (Ag85B) in BCG, called rBCG30 [93]. This vaccine resulted in improved protection against TB in guinea pigs, and appeared to be immunogenic in humans [94]. This vaccine has successfully passed a phase I clinical safety trial but is currently on hold. Following a similar strategy, selected RD1 antigens or larger parts of the RD1 region were transferred into BCG [95]. The rBCG's profile indeed showed better immunogenicity, but this was compromised by its enhanced virulence in SCID mice. Thus, deleting the virulence determinants of this region may offer a viable strategy to improve immunity while maintaining safety. More recent studies engineered a recombinant BCG strain to over-express Ag85A, Ag85B, and TB10.4 [96]. While appearing to be safe and to induce high levels of IFNγ-producing T-cells in mice, its protective efficacy against TB was not improved substantially over wild-type BCG.

Another approach to improve BCG has been the expression of listeriolysin, while deleting expression of ureC in BCG [97]. Listeriolysin is derived from *Listeria monocytogenes*, the organism in which it is required for phagosomal escape and translocation to the cytosol of the infected cell. Listeriolysin likely perforates the phagosomal membrane, allowing leakage of enzymes (such as cathepsins and other proteases) and bacterial components into the cytoplasm of the infected cell. Listeriolysin is only active at acidic pH 5.5 and is degraded immediately in the cytosol because this protein comprises a PEST sequence (i.e., a peptide sequence containing the amino acids proline [P], glutamic acid [E], serine [S], and threonine [T]), which induces its own degradation [98], [99]. Hence, its activity is restricted to the phagosome. These features ensure safety of this construct. BCG normally expresses UreC, which is able to counteract phagosomal acidification; deletion of UreC from BCG prevents this, such that the resulting acidic pH of the BCG phagosome facilitates the activity of listeriolysin. The recombinant BCG lacking ureC and expressing listeriolysin caused superior protection as compared to parental BCG [97]. Interestingly, rBCGΔUrec:Hly appeared to increase apoptosis of infected host cells, and the resulting apoptotic vesicles carrying vaccine components facilitated cross-priming by DCs, inducing strong CD4^+^ and CD8^+^ T-cell responses. In addition, this strain was found to activate Th17-cells, which next to the induced Th1-cells, probably facilitated optimal immunity against *Mtb* [59], [100], [101]. Importantly, this improved rBCG induced superior protection not only against laboratory strains of *Mtb*, but also against clinical *Mtb* isolates with high virulence, including *Mtb* Beijing family strains. The rBCGΔureC:*hly* vaccine VPM1002 has now successfully passed a phase I clinical safety trial and has entered a phase IIa trial in newborns in 2011 (Table 1).

Over-expression of perfringolysin in BCG had rather similar effects as listeriolysin [96]. However, perfringolysin has a relatively broad pH optimum and lacks the PEST sequence [38], such that it can act from inside and outside the cell and remains active in the cytosol. Indeed, a phase I clinical safety trial with rBCG-expressing perfringolysin and Ag85A, Ag85B, as well as antigen Rv3407 (instead of TB10.4) [96], [102], had to be terminated because of side effects (reactivation of shingles [103]) (Aeras-422, see Table 1).

The second class of live *Mtb* vaccines comprises genetically attenuated *Mtb* derivatives. Attenuation can be achieved by inducing auxotrophy, or by deleting essential virulence genes. Auxotrophy relies on deletion of genes involved in the metabolism of essential nutrients for *Mtb*, such as amino acids or pantothenate. The first auxotrophic *Mtb* strains were generated as early as the mid-1990s [104]. More recently, promising candidates for clinical evaluation have been generated, including the *Mtb* ΔRD1ΔpanCD strain, which contains deletions in the major *Mtb* RD1 virulence gene cluster as well as in the biosynthetic pathway of pantothenate [105]. This strain induced efficient protection in normal and immunocompromised mice, and featured promising safety. Another promising attenuated *Mtb* derivative is the ΔPhoPΔfad *Mtb* strain [106]. The two-component transcriptional regulator system PhoP/PhoR regulates the expression of a large number of genes, including *Mtb* virulence genes. The highly attenuated laboratory strain *Mtb* H37Ra was found to contain an inactivating mutation in the *phoP* gene [107]. The *fad* gene is involved in the synthesis of the cell wall lipid phthiocerol dimycocerosate (PDIM). This vaccine candidate is aimed at entering a phase I clinical safety trial in 2012.

### Subunit Vaccines, Primarily Aiming to Boost (Recombinant) BCG-Induced Responses

Subunit vaccines against TB are delivered either through viral vector systems or as recombinant proteins mixed with adjuvants. Two viral delivery systems for TB antigens are currently in clinical trials: Modified vaccinia virus Ankara (MVA) expressing Ag85A has proved extremely immunogenic in both naïve as well as BCG-primed individuals, inducing high Ag85A-specific CD4^+^ T-cell responses in humans [108], [109]. Replication-deficient adenoviral systems have either used Ad5 or Ad35 platforms. Ad35 carrying *Mtb* antigens Ag85A, Ag85B, and TB10.4 was highly immunogenic in humans, inducing strong CD4^+^ and CD8^+^ T-cell and IFNγ responses [110], [111]. Both the MVA over-expressing Ag85A and the Ad35 expressing Ag85A, Ag85B, and TB10.4 are currently in phase IIb clinical trials. The interference of pre-existing human antibodies that could neutralize and negatively impact on adenoviral delivery needs further investigation, but may not be a major impediment to at least some adenoviral vectors [112]. New viral factors are currently being considered, such as replication-defective lymphocytic choriomeningitis virus vectors, which stimulate CD4^+^ and CD8^+^ T-cell as well as B-cell responses [113].

Significant progress has been made in the construction and testing of recombinant proteins, mostly fusion proteins that combine two or more immunodominant antigens of *Mtb*. These are invariably admixed with potent Th1-activating adjuvants. One of the most advanced products is the Hybrid 1 fusion protein, designed by Statens Serum Institute, which consists of Ag85B fused to ESAT6. When administered together with the potent IC31 adjuvant, which activates human Toll-like receptor (TLR)9 and facilitates antigen uptake by DCs, thereby providing a slow-release depot [90], this product leads to strong CD4^+^ Th1 IFNγ induction in humans. This was first reported in naïve human volunteers [114]. An encouraging observation was the long-lasting high-level immune response still detectable 2.5 years after the last vaccination. More recently, the same vaccine was found to boost immune responses previously induced by either BCG or latent *Mtb* infection [115]. The Hybrid 1 fusion protein in IC31 adjuvant is aimed at entering phase IIa trial in 2012. A very similar fusion protein, Hyvac 4, which consists of Ag85B fused to the antigen TB10.4 (which in contrast to ESAT6 is expressed also by BCG), is in a parallel clinical development program (Table 1).

A similar approach was followed by GlaxoSmithKline (GSK) that designed the M72 fusion protein, consisting of *Mtb* antigens Rv1196 and Rv0125 [116]. The M72 fusion protein was admixed with different GSK synthetic adjuvants and had a favorable clinical profile in terms of safety and immunogenicity [116]. The currently favored adjuvant is the AS01_E_, which contains monophosphoryl lipid A, mixed with QS21; the first stimulates TLR4 and drives CD4^+^ Th1 as well as Th17 responses, and the latter primarily triggers CD8^+^ T-cell responses. This vaccine is currently undergoing phase IIa trial. Both AS01_E_ and IC31 are liposomal formulations. Currently other adjuvants are being constructed, such as CAF01 [117], [118]. This liposome-based formulation combines dimethyldioctadecylammonium (DDA) with trehalose 6,6′-dibehenate (TDB), a synthetic analogue of the mycobacterial cord factor trehalose 6,6′-dimycolate (TDM), which ligates the innate immune receptor monocyte-inducible C-type lectin (Mincle) and has strong adjuvant properties [119].

Other antigens close to phase I clinical trial include heparin-binding hemagglutinin (HBHA) [120]. HBHA is expressed by both *Mtb* and BCG and is heavily methylated at its C-terminal portion, against which T-cell responses seem to be specifically directed [121]. The protein's methylation is essential for tethering to and invading human cells, including non-phagocytic cells [122]; this may relate to its ability to facilitate extrapulmonary dissemination of infection. Besides HBHA many other antigens have been discovered that could be interesting vaccine candidates. These include TB latency antigens such as DosR regulon-encoded proteins and starvation antigens like Rv2660, discussed below.

## Designing Better TB Vaccines: The Next Generation

### New Antigens

Until now virtually all TB vaccine strategies have focused on pre-exposure vaccination, which ideally induces strong, long-lasting memory T-cell responses that are rapidly mobilized after infection with *Mtb* and mediate protection. Recent work has revealed that *Mtb* alters its metabolic state from active replication to slow or non-replication during infection. This is accompanied by significant changes in *Mtb*'s gene expression profile. The resulting changes in protein expression also have an impact on which antigens are available to the immune system as a result of *Mtb*'s alterations in antigen expression when entering into starvation or dormancy. Interestingly, this process is partly immune pressure-dependent [123]. The expression of “early secreted antigens" produced by replicating *Mtb* such as Ag85B decrease whereas the expression of “latency antigens" is induced or increased. Such *Mtb* latency antigens are, for example, encoded by (i) the *Mtb* DosR regulon, which mediates *Mtb*'s response to hypoxia; (ii) starvation antigens that are upregulated by *Mtb* upon depletion of nutrients; and possibly also (iii) the broader enduring hypoxic response (EHR) genes, which include many genes of the DosR regulon [124]–[129].

Incorporation of starvation/latency antigens into further improved (live or subunit) vaccines may enhance the impact of these vaccines. The potential success of this approach was recently reported by Peter Andersen and colleagues, who showed that vaccination with a fusion protein linking the Hybrid 1 (Ag85B-ESAT6) backbone to *Mtb* starvation/latency antigen Rv2660c (termed Hybrid 56) induced superior protection against TB, particularly against late chronic infection, when compared to BCG or H1 vaccination. The Rv2660c gene had been selected because of its strong upregulation under starvation conditions [130]. Indeed, this antigen was recognized by T-cells from immune donors in a South African population [131]. H56 was also efficient in preventing TB reactivation in a non-human primate model, in which BCG priming was followed by fusion protein boosting. Even when anti-TNF mAb was used in an attempt to trigger TB reactivation, the vaccine-induced protection remained intact [132]. This vaccine is entering phase I clinical safety testing in 2012.

It is also possible to consider alternative strategies, for example, to include *Mtb* antigens that are expressed by early reactivating bacilli, such as resuscitation-promoting factor antigens or downstream antigens [75], [102], [126]. The availability of a pool of memory T-cells able to respond to these rapidly expressed reactivation antigens might provide a means of promptly detecting and controlling reactivated *Mtb*.

### The Complex Milieu of the Vulnerable Human Host: Immune Modulation by External, Environmental, and Endogenous Factors

In real life, *Mtb* infection occurs in individuals who are often co-infected by environmental microbes or pathogens that can modulate immune responses, including responses to vaccination such as BCG vaccination. Non-tuberculous (environmental) mycobacteria (NTM) can significantly downregulate and alter the profile of BCG-induced immune responses [15], [133]. Nutritional factors such as iron status and vitamin D, which influences innate immunity by inducing anti-microbial peptides, such as cathelicidin (LL-37) and autophagy in association with IFNγ, also impact TB host immunity [134], [135]. Different *Mtb* lineages or genetic differences in virulence between *Mtb* strains are also important in this context. Clinical programs will need to compare vaccination efficacy in different geographic and ethnic settings, and evaluate the impact of differences in *Mtb* epidemiology and strains or clades on vaccine-induced immune responses and protective efficacy [1].

*Mtb* itself is a master regulator of immune regulatory circuits. It induces strong CD4^+^ and CD8^+^ Treg activity that suppresses Th1 and Th17 responses (see above). *Mtb* exploits these regulatory mechanisms, which are normally used to prevent excessive inflammatory responses and accompanying tissue damage. In addition, *Mtb* downregulates key pathways of protection such as apoptosis, autophagy, and IFNγ production as well as IFNγR signaling. Of note, highly discriminative biomarkers upregulated in T lymphocytes from TB patients as compared to latently infected individuals comprise genes involved in inhibitory signaling [136]. The likely synergistic effects of blocking multiple pathways at multiple different levels, added to the delay in its transport from the lung to the lymph node and the ensuing delay in priming T-cell immunity, collectively create an environment in which *Mtb* can establish a safe niche of infection from which it can seed the human body with a new wave of bacteria whenever immune pressure is alleviated, for example, during co-infection or malnutrition. Thus, the challenge for new TB vaccines is not only to induce strong memory immune responses with long-term efficacy in “clean animal laboratory settings", but also to do so in the face of the highly complex environmental, co-infection, and immunoregulatory background of the vulnerable human host.

### Therapeutic Vaccines

New TB vaccines should not only induce immune responses that control existing or new infections at the latent stage, but ideally also eradicate *Mtb* organisms from the human body and achieve sterile eradication [25]. This would avoid the threat of TB reactivation from latency that can occur decades after initial infection. Such vaccines must also operate in the huge reservoir of latently infected individuals. Vaccines considered to be useful in such settings need to express antigens that are expressed by *Mtb* organisms during latent-stage infection. A major unknown is the precise mechanism that mediates protective immunity and possibly sterile eradication of *Mtb*. Recent results obtained with the above-discussed modified *M. smegmatis* vaccine are promising [92] with respect to the latter. However, in the absence of detailed knowledge, it would seem fortuitous to develop vaccines that stimulate all arms of the immune system, including Th1 cells; Th17 cells (that play a role in the early response, possibly through the recruitment of neutrophils); CD8^+^ T-cell populations with cytolytic and tuberculocidal activity (e.g., through the activity of perforin and granulysin; apoptosis; autophagy), as well as TCRγδ cells and NK T cells, which bridge innate and adaptive immunity, and provide IL-17 and IFNγ early in the response to infection [137]; and perhaps, lipid reactive T-cells, notably CD1-restricted T-cells that can express a myriad of relevant effector functions against *Mtb*.

### A Role for B-Cells and Fc Receptors?

Vaccines that prevent productive *Mtb* infection are needed. Only very few bacilli are thought to reach the lung and establish the primary complex. Theoretically, the availability of mucosal antibodies at the site of infection could be valuable. It is interesting to consider that recent studies have documented a hitherto unappreciated role for B-cells in potentiating protective immunity against *Mtb* [76]. What is the potential role of such antibodies? First, they could target *Mtb* to macrophages for destruction, e.g., through FcγR pathway activation. In this context it is interesting to note that FcγR is among the most discriminative markers upregulated in latently infected individuals as compared to TB patients [78], [138]. Second, they might exert chemotactic functions and attract additional immune cells such as monocytes, neutrophils, and NK cells to the site of infection. And third, by targeting *Mtb* to Fc receptors, antibodies might promote activation and antigen processing/presentation by DCs and macrophages and promote microbicidal activity in these cells [139], [140].

Other TB vaccination strategies should address the targeting of the mucosal immune system. Much research is focusing on the potential of mucosal immunity and its enhancement by local vaccine delivery. Recent studies documented that pulmonary aerosol delivery of BCG or subunit vaccines in animal models was highly effective in inducing rapid immunity at the site of infection [141]–[144]. Exploring new routes of vaccination and targeting unique mucosal immune response pathways, which include mucosal cells such as intraepithelial cells, MAIT, and T-cells expressing homing receptors for the lung, such as CXCR6 [145], have been relatively unexplored in TB and may have high potential. Even though such cells are unlikely to prevent TB infection *stricto sensu*, their rapid early response is likely to contribute to early control of infection, particularly because these cells may respond to infection during a critical period in which *Mtb* delays onset of adaptive immunity as discussed above.

## Concluding Remarks

In striking contrast to only 10 years ago, the TB vaccine candidate pipeline is now filled with a number of high-profile candidates, some of which have already entered or are soon likely to enter clinical trials. First immunogenicity profiles of vaccines tested in humans look encouraging. Because we do not know the mechanisms of protection against TB and are lacking surrogate end-point biomarkers of protection, we cannot predict whether these vaccines will have protective efficacy in humans. Nevertheless, many of these vaccine candidates have proven records in animal models, ranging from mice to guinea pigs to non-human primates, raising hopes they may also be protective in humans. Vaccines may be used either as stand-alone vaccines or—more likely—in heterologous prime-boost settings.

The next 10–15 years will be critical for TB vaccines to demonstrate protective efficacy as well as safety. At the same time, it must be realized that these first-generation vaccines are neither designed to prevent infection nor to achieve sterile eradication, but rather to better prime and/or boost infection control. Thus at best, such vaccines will only inhibit or delay TB reactivation. Next-generation vaccines should be designed aiming at preventing infection or achieving sterile eradication, perhaps by redirecting immune responses at *Mtb* antigens expressed during latency [124]–[126], [130] or by using new platforms [92]. The new vaccines, especially subunit vaccines, most likely will need to be administered multiple times in order to maintain adequate life-long immune memory. Virtually nothing is known about the timing of such boosting schedules when it comes to vaccines aimed at inducing primarily cellular immunity. Finally, the impact of co-infection, immune regulation, and environmental factors (nutrition; type-2 diabetes) that enhance susceptibility to developing TB disease remain ill-defined, and need further research.

In summary, there is reason for optimism in the field of TB vaccines: there is a substantial portfolio of interesting TB vaccine candidates in clinical phase-I/II testing, some of which are already fairly advanced in the TB vaccine pipeline. This is complemented by numerous promising candidates further upstream in the pipeline. In fact, an emerging bottleneck may not be the number of pre-clinical TB vaccine candidates that TB researchers can produce, but rather the number of vaccines that can be tested clinically in efficacy trials, given the limited clinical trial capacity worldwide, i.e., a shortage that exists not only in Africa but also in Asia [1], [3]. Finally, the identification of TB surrogate end-point biomarkers or “correlates of protection" may drastically reduce the need for the current long-term large-scale clinical trials, and thus will speed up TB vaccine discovery and clinical testing [1], [9].
